# The predictive value of eosinophil levels on no-reflow in patients with STEMI following PCI: a retrospective cohort study

**DOI:** 10.1038/s41598-022-22988-2

**Published:** 2022-10-25

**Authors:** De-Gang Mo, Chun-Song Wang, Jia-Hui Liu, Tai Li

**Affiliations:** 1 Department of Cardiology, Shandong First Medical University, Jinan, 250118 People’s Republic of China; 2 Department of Cardiology, Liaocheng People’s Hospital Affiliated to Shandong First Medical University, Liaocheng, 252000 People’s Republic of China; 3 Department of Nursing, Liaocheng Vocational and Technical College, Liaocheng, 252000 People’s Republic of China

**Keywords:** Biomarkers, Cardiology, Medical research

## Abstract

In patients with acute ST-elevation myocardial infarction (STEMI), it is essential to restore myocardial perfusion as soon as possible. However, a considerable proportion of patients have no-reflow. No-reflow increases the risk of major adverse cardiac events and even death. The role of blood eosinophil count in predicting no-reflow in STEMI patients has not been determined, particularly after primary percutaneous coronary intervention (pPCI). The present study aimed to evaluate the predictive value of eosinophil counts for no-reflow in patients with STEMI who underwent pPCI. A total of 674 STEMI patients who underwent pPCI were enrolled. The subjects were divided into two groups according to eosinophil counts for primary analysis and with or without T_2_DM for secondary analysis. Logistic regression analysis was used to determine whether eosinophil count was an independent predictor of no-reflow in the entire cohort, and subgroup and receiver operating characteristic (ROC) curves were explored to evaluate its predictive value. DeLong’s test was used to compare the area under curves of the three ROC curves. The low eosinophil count was an independent predictor for no-reflow in whole cohort (adjusted OR: 2.012, 95% CI 1.242–3.259, *p* = 0.004) and in patients with T_2_DM (adjusted OR: 4.312, 95% CI 1.878–9.900, *p* = 0.001). In patients without T_2_DM, hemoglobin, but not low eosinophil count, was an independent predictor of no-reflow. The results of the ROC curve analysis revealed that a low eosinophil count had moderate predictive efficiency for predicting no-reflow in patients with T_2_DM, and the power was superior to all populations and patients without T_2_DM. Our data suggest that decreased eosinophil count was an independent risk factor for no-reflow in patients with STEMI who underwent pPCI, especially in T_2_DM patients, which provides guidance for clinicians to identify patients at a higher risk of developing no-reflow and lowering their risk.

## Introduction

Myocardial perfusion should be restored as soon as possible in patients with acute ST-elevation myocardial infarction (STEMI)^1^. Primary percutaneous coronary intervention (pPCI) to achieve a resumption of optimal blood flow is the preferred method of reperfusion, which significantly prevents further necrosis of the myocardium and improves the quality of life of patients with acute myocardial infarction (AMI)^2,3^. However, a considerable proportion of patients who undergo pPCI have no-reflow, which can lead to adverse left ventricular remodeling and poor healing of the infarct, increasing the risk of major adverse cardiac events (MACE) and even death^4,5^. The impact of no-reflow on clinical outcomes has been well documented, and the incidence of no-reflow ranges from 2 to 60%^6,7^. Although no-reflow has been intensively studied, its detailed molecular mechanisms remain unclear^8,9^.

Accumulating evidence showed that inflammation was pivotal in developing no-reflow^10,11^. Many studies have evaluated the involvement of classic inflammatory responses in no-reflow, such as neutrophils, monocytes/macrophages, and T cells^12–14^. A previous study showed that C-reactive protein (CRP), albumin, white blood cell count (WBC) count, neutrophil-to-lymphocyte ratio (NLR), and CRP/albumin ratio (CAR) can be used to predict no-reflow in patients with STEMI who treated with pPCI. Moreover, the authors also found that the CAR has a stronger prediction ability than that of CRP, WBC, and NLR^15^. However, in addition to classic inflammation, both experimental and clinical studies suggest that allergic inflammation is also involved in the pathogenesis of coronary artery disease (CAD) and MACE following stent implantation^16–18^. Moreover, allergic inflammation, largely mediated by eosinophils, has recently been found to contribute to atherosclerotic plaque formation and thrombosis^18^. Previous studies have shown that eosinophil levels emerged as a strong predictor of mortality in patients with CAD undergoing PCI^19^, In fact, previous studies have unearthed that eosinophils levels emerged as a strong predictor of mortality in patients with CAD undergoing PCI^20^, and in patients with acute heart failure (HF)^21^. The role of blood eosinophil counts in predicting no-reflow in patients with STEMI, particularly following pPCI, has not been determined.

In light of the above, we aimed to assess the possible relationship between blood eosinophils count and no-flow in patients with STEMI who are undergoing pPCI. In addition, we studied their relationship in a subgroup of patients with and without T_2_DM.

## Methods

### Study design and patient selection

**Figure 1:**
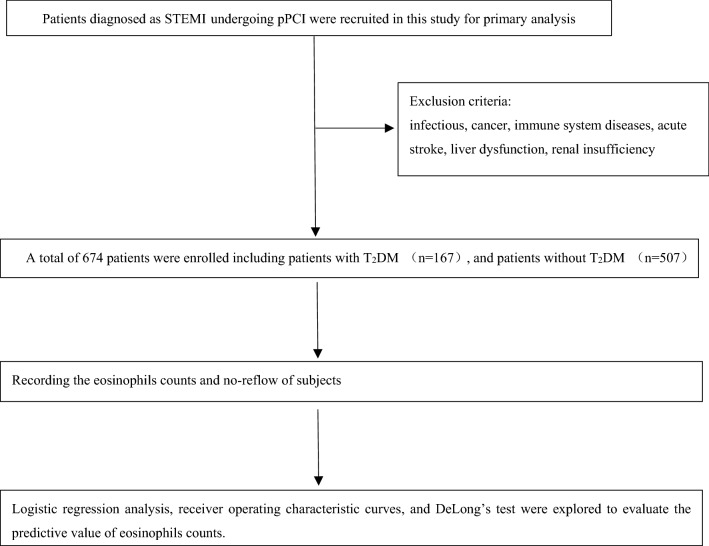
The flowchart of studied patients.

This was a single-center retrospective observational cohort study. Patients with STEMI who underwent pPCI at the Liaocheng People’s Hospital between June 2016 and November 2019 were enrolled in this study. ESC guidelines for managing STEMI were defined as diagnostic criteria^22^. The inclusion criteria were STEMI patients who underwent pPCI with stent implantation or percutaneous transluminal coronary angioplasty. Patients hospitalized for STEMI but with severe infections, severe renal and liver diseases, immune system diseases, aortic dissection, or cancer were excluded. Ultimately, 674 patients were included in the analysis. The study flowchart is shown in Fig. 1. All protocols were approved by the ethics committee of Liaocheng People’s Hospital. As this was a retrospective study, informed consent forms were exempted from the ethics committee of Liaocheng People’s Hospital.

### Study variables and laboratory testing

Clinical characteristics and demographic profiles were obtained from the hospital’s computerized information system. All venous blood samples were collected from patients before the pPCI procedure at admission. All laboratory tests were performed at our hospital emergency laboratory. Whole-blood eosinophils counts (reference range: 0.02–0.52 × 10^9^/L) were determined on a CELL DYN 4000 Abbott analyzer (Abbott Diagnostics, Santa Clara, California, USA), which was calibrated daily; and 0.02–0.52 × 10^9^/L was defined as normal.

### Primary PCI and no-reflow

Upon admission, all patients received a standard loading dose of medications (300 mg aspirin and 180 mg ticagrelor or 300 mg clopidogrel) upon the diagnosis of STEMI. A total of 2500 IU of heparin was administered, and a weight-dependent dose (up to 100 IU/kg) was added for PCI. All surgical procedures and decisions were made by experienced cardiologists. No-reflow phenomenon was defined according to coronary thrombolysis in myocardial infarction (TIMI) flow, and TIMI flow grade of 0–II after vessel reopening without coronary stenosis, dissection, and spasm was defined as “no-reflow.”

### Statistical analysis

Statistical analysis was performed using SPSS 23.0 (IBM Corp.) and MedCalc Statistical Software, version 16.8.4 (Ostend, Belgium.). Normally distributed continuous data were expressed as mean ± standard deviation and were compared using the Student’s *t*-test. Non-normally distributed data were expressed as median (interquartile range) and compared using the Mann–Whitney U test. Categorical variables are expressed as frequencies (percentages) and compared using the chi-square or Fisher’s exact test. Logistic regression analysis was used to determine whether eosinophil count was an independent predictor of no-reflow in the total study population and in patients with and without T_2_DM. Variables with an unadjusted *p* value < 0.05 in the univariate analysis were subsequently evaluated using a multivariate logistic regression model. Receiver operating characteristic (ROC) curves were used to determine the best cut-off values of eosinophil count for predicting no-reflow in the study population and patients with and without T_2_DM, respectively. DeLong’s test was used to compare area under curves (AUCs) of the three ROC curves. A *p* value < 0.05 was the criteria for statistical significance in this analysis.

### Ethics approval and consent to participate

This study was approved by the Medical Ethics Committee of Liaocheng People’s Hospital. All procedures were in accordance with principles of Helsinki Declaration. Since it is a retrospective study, informed consent forms were exempted by the Ethics Committee of the Liaocheng People’s Hospital.

## Results

### Patient characteristics

Between June 2016 and November 2019, 674 patients who underwent pPCI were included in this study. Of the 674 patients, 455 patients (67.5%) had normal eosinophil counts (≥ 0.02 × 10^9^/L) and 219 patients (32.5%) had decreased eosinophil counts (< 0.02 × 10^9^/L). The baseline characteristics of the two groups are shown in Table 1. Patients who had decreased eosinophil counts were nonsmokers (*p* < 0.001), had higher levels of white blood cells (*p* < 0.001) and D-dimer (*p* = 0.026), and a higher incidence of no-reflow (*p* = 0.001).

**Table 1:** Basic characteristics of patients by eosinophils counts.

Variable	Eosinophils counts < 0.02 × 10^9^/L	Eosinophils counts ≥ 0.02 × 10^9^/L	*p* Value
	n = 219	n = 455	
Age (year)	62 (16)	61 (18)	0.688
Male, n (%)	143 (65.3)	361 (79.5)	< 0.001
Smoking, n (%)	96 (43.8)	272 (59.9)	< 0.001
Hypertension, n (%)	111 (50.6)	252 (55.5)	0.252
T_2_DM, n (%)	57 (26.0)	110 (24.2)	0.602
Heart rate (bpm)	78 (21)	76 (20)	0.062
Hemoglobin (g/dL)	141 (24)	144 (20)	0.137
WBC count (× 10^9^/L)	10.46 (3.90)	9.47 (3.98)	< 0.001
Eosnophils count (× 10^9^/L)	0.01 (0.01)	0.06 (0.09)	< 0.001
PLT (× 10^9^/L)	227 (73)	231 (83)	0.394
D-Dimer (ng/ml)	0.40 (0.60)	0.30 (0.41)	0.026
Cr (umol/L)	60 (20)	64 (20)	0.005
TC (mmol/L)	4.73 (1.27)	4.74 ± 1.33	0.393
TG (mmol/L)	1.20 (1.08)	1.54 (1.17)	< 0.001
LV (mm)	46 (6)	46 (6)	0.938
LVEF (%)	50 (9)	50 (10)	0.025
Clopidogrel, n (%)	58 (26.5)	74 (16.3)	0.002
Ticagrelor, n (%)	161 (73.5)	381 (83.7)	0.002
No-reflow, n (%)	41 (18.7)	43 (9.5)	0.001

*T2DM* type 2 diabetes mellitus, *WBC* white blood cell, *PLT* platelet count, *Cr* creatinine, *TC* total cholesterol, *TG* total glyceride, *LV* left ventricular thickness, *LVEF* left ventricular ejection fraction.

In this study, the patients were divided into two groups for further analysis depending on the presence or absence of T_2_DM: 167 (24.8%) patients with T_2_DM and 507 (75.2%) patients without T_2_DM; the results are shown in Table 2. Patients with T_2_DM were male (*p* = 0.004), non-smokers (*p* < 0.001), had a higher triglyceride level (*p* = 0.012), and a higher incidence of no-reflow (*p* < 0.001).

**Table 2:** Basic characteristics of patients with or without T_2_DM.

Variable	All patients	Without T_2_DM	With T_2_DM	*p* Value
	n = 674	n = 507	n = 167	
Age (year)	62 (17)	61 (17)	63 (16)	0.242
Male, n (%)	504 (74.8)	393 (77.5)	111 (66.5)	0.004
Smoking, n (%)	368 (54.5)	297 (58.6)	71 (42.5)	< 0.001
Hypertension, n (%)	363 (53.9)	268 (52.9)	95 (56.9)	0.365
Heart rate (bpm)	76 (21)	76 (20)	77.89 ± 15.84	0.421
Hemoglobin (g/dL)	143 (21)	144 (22)	140.97 ± 16.69	0.136
WBC count (× 10^9^/L)	9.95 (4.00)	9.96 (4.00)	9.83 (4.06)	0.507
Eosnophils count (× 10^9^/L)	0.03 (0.08)	0.03 (0.07)	0.03 (0.08)	0.675
PLT (× 10^9^/L)	229 (79)	230 (80)	228 (72)	0.937
D-Dimer (ng/ml)	0.32 (0.44)	0.31 (0.50)	0.32 (0.43)	0.711
Cr (umol/L)	63 (20)	64 (20)	59 (22)	< 0.001
TC (mmol/L)	4.74 (1.29)	4.75 (1.26)	4.73 (1.41)	0.930
TG (mmol/L)	1.46 (1.19)	1.41 (1.13)	1.55 (1.17)	0.012
LV (mm)	46 (6)	46 (6)	46 (6)	0.053
LVEF (%)	50 (10)	50 (10)	51 ± 11	0.164
Clopidogrel, n (%)	132 (19.6)	100 (19.7)	32 (19.2)	0.874
Ticagrelor, n (%)	542 (80.4)	407 (80.3)	135 (80.8)	0.874
No-reflow, n (%)	84 (12.5)	50 (9.9)	34 (20.3)	< 0.001

*T*_*2*_*DM* type 2 diabetes mellitus, *WBC* white blood cell, *PLT* platelet count, *Cr* creatinine, *TC* total cholesterol, *TG* total glyceride, *LV* left ventricular thickness, *LVEF* left ventricular ejection fraction.

### The results of multivariate logistic regression analysis

For predicting no-reflow in the overall population, the multivariate logistic regression model, including variables such as age, sex, smoking status, T_2_DM, and low eosinophil count, showed that low eosinophil count was an independent predictor for no-reflow in the overall population (adjusted OR: 2.012, 95% CI 1.242–3.259, *p* = 0.004). Variables such as smoking status, T_2_DM, and hemoglobin level were independent factors for no-reflow (Table 3).

**Table 3:** Logistic regression analysis of predictors of no-reflow.

Variables			OR	95% CI	*p* Value	Adjusted OR	95% CI	*p* Value
Age		A	1.027	1.006–1.048	0.013	1.014	0.991–1.038	0.225
		B	1.014	0.978–1.050	0.455			
		C	1.033	1.006–1.060	0.017	1.015	0.987–1.044	0.291
Gender		A	2.016	1.247–3.257	0.004	1.300	0.690–2.449	0.416
		B	1.772	0.820–3.828	0.146			
		C	1.912	1.022–3.578	0.043	1.286	0.557–2.972	0.556
Smoking		A	2.561	1.587–4.133	< 0.001	1.914	1.074–3.409	0.028
		B	3.578	1.457–8.784	0.005	3.249	1.266–8.334	0.014
		C	1.923	1.067–3.466	0.030	1.535	0.737–3.196	0.252
Hypertension		A	1.101	0.695–1.745	0.681			
		B	0.477	0.212–1.075	0.074	0.391	0.162–0.946	0.037
		C	1.357	0.755–2.437	0.307			
T2DM		A	2.337	1.451–3.763	< 0.001	2.136	1.303–3.500	0.003
Heart rate		A	0.995	0.981–1.009	0.505			
		B	1.005	0.982–1.029	0.663			
		C	0.988	0.970–1.006	0.193			
Hemoglobin		A	0.977	0.965–0.990	< 0.001	0.984	0.969–0.999	0.039
		B	0.996	0.973–1.018	0.703			
		C	0.969	0.953–0.984	< 0.001	0.972	0.955–0.990	0.002
WBC count		A	1.048	0.979–1.122	0.178			
		B	1.088	0.981–1.207	0.111			
		C	1.021	0.930–1.120	0.663			
Low eosinophils		A	2.207	1.390–3.505	0.001	2.012	1.242–3.259	0.004
		B	4.353	1.975–9.594	< 0.001	4.312	1.878–9.900	0.001
		C	0.103	0.002–6.780	0.287			
PLT		A	0.999	0.996–1.003	0.742			
		B	1.004	0.998–1.010	0.182			
		C	0.996	0.991–1.001	0.157			
Cr		A	0.991	0.977–1.005	0.206			
		B	0.994	0.974–1.014	0.565			
		C	0.993	0.975–1.012	0.495			
TC		A	0.967	0.779–1.201	0.761			
		B	0.928	0.678–1.272	0.644			
		C	1.003	0.752–1.338	0.981			
TG		A	0.956	0.800–1.143	0.620			
		B	0.990	0.796–1.232	0.927			
		C	0.823	0.609–1.113	0.206			
D-dimer		A	1.146	0.947–1.387	0.162			
		B	1.194	0.843–1.691	0.319			
		C	1.231	0.733–1.752	0.324			

group A: all patients B: patients with T_2_DM C: patients without T_2_DM.

*OR* odds ratio, *CI* confidence interval, *T*_*2*_*DM* type 2 diabetes mellitus, *WBC* white blood cell, *PLT* platelet count, *Cr* creatinine, *TC* total cholesterol, *TG* total glyceride.

In patients with T_2_DM, the variables in the multivariate logistic regression model were smoking status, history of hypertension, and low eosinophil count. The results revealed that low eosinophil count was an independent predictor of no-reflow in the T_2_DM population (adjusted OR: 4.312, 95% CI 1.878–9.900, *p* = 0.001) (Table 3).

In patients without T_2_DM, the variables in the multivariate logistic regression model were age, smoking status, sex, and hemoglobin level. The results revealed that hemoglobin, but not low eosinophil count, was an independent predictor of no-reflow in patients without T_2_DM (adjusted OR: 0.972, 95% CI 0.955–0.990, *p* = 0.002) (Table 3).

### The results of ROC curves

**Figure 2:**
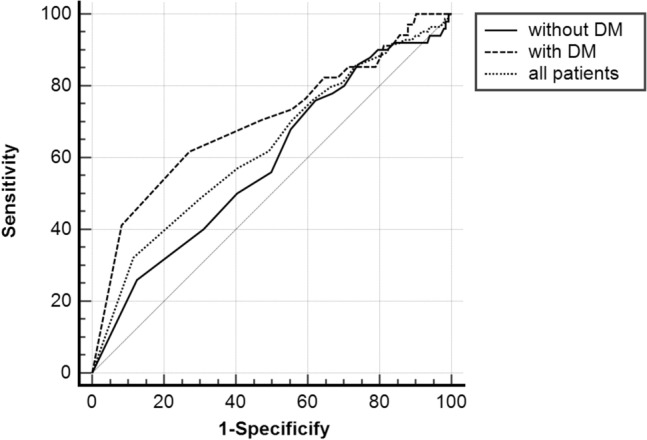
The ROC curves in predicting no-reflow.

ROC curves were generated to evaluate the potential predictive power of low eosinophil count for no-reflow (Fig. 2). The results of the ROC curve analysis revealed that a low eosinophil count had moderate predictive efficiency for predicting no-reflow in patients with T_2_DM, and the power was superior to all populations and patients without T_2_DM. The performances of low eosinophil count in predicting no-reflow are shown in Table 4. DeLong’s test was used to compare the area under curves (AUCs) of the three ROC curves. It showed that there was no significant difference between the three ROC curves (Table 5).

**Table 4:** The performance of low eosinophil counts in predicting no-reflow.

	AUC	95% CI	*p* Value	Sensitivity (%)	Specificity (%)	Cut-off
Total	0.627	0.560–0.694	< 0.001	68	88	0.005
With T_2_DM	0.700	0.592–0.808	< 0.001	62	73	0.015
Without T_2_DM	0.585	0.500–0.670	0.048	76	38	0.055

*T*_*2*_*DM* type 2 diabetes mellitus, *AUC* area under curve, *CI* confidence interval.

**Table 5:** Paired comparison of ROC curves (DeLong’s test).

	Difference of AUC	Standard error	95% CI	Z value	*p* Value
0 versus 1	0.115	0.0702	− 0.0225–0.253	1.640	0.1010
0 versus 2	0.042	0.0552	− 0.0664–0.150	0.757	0.4493
1 versus 2	0.073	0.0650	− 0.0540–0.201	1.129	0.2587

0: patients without T_2_DM; 1: patients with T_2_DM; 2: all patients.

*T2DM* type 2 diabetes mellitus, *AUC* area under curve, *CI* confidence interval.

## Discussion

In this retrospective study, eosinophil count was an independent predictor of no-reflow in patients with STEMI who underwent pPCI. In the subgroup of patients with T_2_DM, eosinophil level was an independent risk factor for no-reflow, but not in patients without T_2_DM.

An increasing number of studies have demonstrated the value of plasma eosinophil counts in patients with CAD. However, the role of eosinophils in CAD remains unclear. Gao et al.^20^ revealed that the percentage of eosinophils was lower in patients with CAD, especially in those with AMI. Furthermore, low eosinophil count was strongly associated with severe CAD and acute coronary arterial thrombotic events. Jiang et al.^23^ showed that patients with AMI presenting with decreased eosinophil counts had serious myocardial damage. This study indicated that eosinophils play an important role in thrombosis in patients with CAD. Data from the CALIBER study showed a strong correlation between low eosinophil count, HF, and death^24^. In a prospective series of 620 patients with STEMI, lower minimum eosinophil counts were associated with more extensive edema, microvascular obstruction, infarct size, and a higher rate of cardiac events (death, reinfarction, or heart failure) during follow-up^25^. In a recent animal study, an increase in heart and blood eosinophils post-MI represented a compensatory mechanism to protect the heart from ischemic injury^26^. Furthermore, genetic and pharmacological eosinophil depletion leads to increased adverse remodeling in experimental AMI^27^. These results indicate that a higher eosinophil count is protective in patients with CAD. However, other studies have suggested a destructive effect of eosinophils on CAD. Increased eosinophils were an independent predictor of death in 8943 consecutive patients with triple-vessel CAD after a median of 7.5 years of follow-up^28^. A high preprocedural eosinophil count was associated with improved outcomes within the first 6 months; however, after this period, there was an increased risk of mortality^29^. Therefore, the value of eosinophils in CAD remains controversial.

However, the association between eosinophil levels and no-reflow was not explored in the studies above. Briefly, the present study results followed the findings of previous studies, and our study is the first to reveal an association in the pPCI population and further elucidate the T_2_DM-related difference in plasma eosinophil count predicting no-reflow. Further analysis with a larger population sample might help clarify the role of plasma eosinophil count in patients with CAD, especially in predicting no-reflow. A previous study showed that eosinophils contribute to atherosclerotic plaque formation and thrombosis through their interplay with platelets. Additionally, they found high numbers of eosinophils in coronary artery thrombi, and female patients with stent thrombosis had the highest eosinophil counts^19^. Therefore, we speculated that the eosinophil count was decreased in the peripheral blood. Mechanisms that explain the value of eosinophil counts in predicting the incidence of no-reflow in patients with STEMI are warranted. In our study, DeLong’s test show that there was no significant difference between the three ROC curves. This may be due to the small sample size. Multi center research with large sample size is needed in future research.

This study has several limitations. This was a single-center study, and plasma eosinophil count was not dynamically monitored. Moreover, our study did not collect data such as the total ischemic time, culprit lesion characteristics, and procedural details. Finally, further studies are required to better understand the pathophysiological role of eosinophils and to explore the potential therapeutic implications.

## Conclusions

In this retrospective cohort study, a decreased plasma eosinophil count was an independent risk factor for no-reflow in patients with STEMI who underwent pPCI, especially in T_2_DM patients. This analysis highlighted the importance of eosinophil count and guided clinicians in identifying patients at a high risk of developing no-reflow and lowering their risk.

## Data Availability

The datasets used and/or analysed during the current study are available from the corresponding author on reasonable request.

## Abbreviations

STEMI: ST-segment elevation myocardial infarction
pPCI: Primary percutaneous coronary intervention
T_2_DM: Type 2 diabetes mellitus
ROC: Receiver operating characteristic
AUC: Area under curves
OR: Adjusted odds ratio
CI: Confidence interval
AMI: Acute myocardial infarction
MACE: Major adverse cardiac events
CRP: C-reactive protein
WBC: White blood cell count
NLR: Neutrophil-to-lymphocyte ratio
CAR: CRP/albumin ratio
CAD: Coronary artery disease
TIMI: Thrombolysis in myocardial infarction
TG: Total glyceride
